# Trichinellosis Outbreak Linked to Undercooked Bear Jerky, North Carolina, USA, 2024

**DOI:** 10.3201/eid3207.260062

**Published:** 2026-07

**Authors:** Camden D. Gowler, Nicole Lee, Meghan Phillips, Sarah G.H. Sapp, Tammra Morrison, Melanie D’Angelo, Margaret Neja, Billy A. Watson, Susan P. Montgomery, Anne Straily, Carl Williams, Erica Wilson

**Affiliations:** North Carolina Department of Health and Human Services, Raleigh, North Carolina, USA (C.D. Gowler, N. Lee, T. Morrison, M. D’Angelo, C. Williams, E. Wilson); Centers for Disease Control and Prevention, Atlanta, Georgia, USA (C.D. Gowler, S.G.H. Sapp, M. Neja, B.A. Watson, S.P. Montgomery, A. Straily); Graham County Department of Public Health, Robbinsville, North Carolina, USA (M. Phillips)

**Keywords:** Trichinellosis, Trichinella, *Trichinella spiralis*, North Carolina, parasites, black bears, serology, albendazole, United States

## Abstract

*Trichinella* spp. nematodes are parasites that can cause trichinellosis in humans after consumption of infected, undercooked meat. A November 2024 trichinellosis outbreak in western North Carolina, USA, resulted in 3 cases (2 probable, 1 confirmed), all linked to undercooked bear jerky. In total, 6 persons consumed the implicated meat (attack rate 50%). Molecular testing identified *Trichinella spiralis* in leftover meat from the same bear. This outbreak provides evidence of changing trichinellosis patterns. Low-cost safety measures and prevention efforts regarding safe wild game preparation are needed to avoid future outbreaks.

*Trichinella* spp. are parasitic nematodes that can cause trichinellosis (also called trichinosis) when humans consume infected, undercooked meat. Trichinellosis is a reportable disease in North Carolina, USA. Only 3 cases were reported during 1991–2022, and no outbreaks were reported during the same period. Historical reports suggest very low incidence locally (*1*,*2*). In November 2023, a probable trichinellosis outbreak was reported, and undercooked bear meat was the likely source (*3*). A year later, in November 2024, a public health investigation was initiated when a clinician reported a hospitalized patient with suspected trichinellosis. This patient had shared wild bear meat with 5 other persons in the form of jerky (desiccated meat). The Graham County Health Department and North Carolina Division of Public Health (NCDPH) investigated to characterize cases and provide public health guidance to prevent further illness.

## Methods

The week after the November 2024 report, public health officials identified and interviewed 6 persons who consumed the bear jerky. We used the 2014 case definition from the Council for State and Territorial Epidemiologists (*4*) for case classification. We defined probable cases as the presence of clinically compatible symptoms in a person who shared an epidemiologically implicated meal or ate a meat product in which the parasite was found. We defined confirmed cases as the presence of clinically compatible symptoms with a positive laboratory test such as *Trichinella* antibody screening. Clinically compatible signs and symptoms included fever, myalgia, periorbital edema, and eosinophilia. This activity was reviewed by the Centers for Disease Control and Prevention (CDC), was deemed not research, and was conducted consistent with applicable federal law and CDC policy.

The bear was hunted in Graham County in western North Carolina at the start of bear season (October). Approximately half the meat was frozen as various cuts and half was prepared as jerky without prior freezing. Because the process involved only marination and dehydration, the jerky preparation likely did not reach the suggested >165°F (>74°C) internal temperature necessary to kill *Trichinella* spp. parasites. At the time of notification, purportedly no jerky remained, but 4 remaining frozen meat pieces were sent for testing at the CDC’s Division of Parasitic Diseases and Malaria, National Center for Emerging and Zoonotic Infectious Diseases.

The implicated bear jerky was shared among 6 persons. Trichinellosis symptoms developed in 3 persons, including 1 who was hospitalized with severe symptoms characteristic of trichinellosis: periorbital edema, eosinophilia, and muscle weakness. The patient’s physician had read of an outbreak of trichinellosis in western North Carolina reported in Morbidity and Mortality Weekly Report (*3*) and informed NCDPH. Public health officials advised those involved to cease further jerky consumption. State wildlife officials emailed a notice to a listserv of registered hunters about proper handling and cooking of bear meat.

## Results

The overall attack rate was 50% (n = 3), including probable cases in persons who ate the implicated meal and reported symptoms (Table 1). For confirmed cases only, the attack rate was 16% (n = 1). Median incubation period for symptomatic cases was 33 (range 27−40) days. The hospitalized patient was tested for *Trichinella* IgG twice. The first results were negative (collected 12 days after symptom development), but convalescent serum results (collected 31 days after symptom development) were positive (Table 2). Public health officials advised the remaining symptomatic persons of the value of testing, including convalescent testing, because of potential early false negatives. However, because they did not have health insurance, both were discouraged by the high cost (>$200) of testing. All symptomatic persons were treated with albendazole and recovered (Table 1).

**Table 1:** Case classification, demographics, and symptoms for all persons who consumed undercooked bear jerky in trichinellosis outbreak in North Carolina, USA, 2024 *

Characteristics	Age grouping					
	20–29	50–59	20–29	30–39	20–29	Unknown
Sex	M	M	F	M	M	M
Classification	Confirmed	Probable	Probable	Not a case	Not a case	Not a case
Symptomatic	Yes	Yes	Yes	No	No	No
Hospitalization	Yes	No	No	NA	NA	NA
Treatment†	Yes	Yes	Yes	NA	NA	NA
Symptom onset, days	25	38	31	NA	NA	NA
Eosinophil count, K/µL (%)‡	3.69 (22)	Not performed	Not performed	NA	NA	NA
Symptoms	Fever, myalgia, photophobia, muscle weakness, periorbital edema, subungual and retinal hemorrhage, elevated CRP, eosinophilia	Fever, myalgia	Myalgia	NA	NA	NA

*CRP, C-reactive protein; NA, not applicable. †Primary treatment was albendazole. ‡Reference range 0.00–0.50 K/µL (0.0%–7.0%).

**Table 2:** Trichinella antibody testing timeline and results from patient who consumed undercooked bear jerky in trichinellosis outbreak in North Carolina, USA, 2024

Age group	Sex	Classification	First IgG test timepoint	First IgG test result	Second IgG test timepoint	Second IgG test result
20–29	M	Confirmed	37 days after meal*	Negative	56 days after meal*	Positive

*On the basis of initial consumption of bear jerky.

CDC’s Division of Parasitic Diseases and Malaria tested 4 bear meat specimens. The meat was frozen at an unknown temperature for >57 days. Testing found encapsulated *Trichinella* spp. larvae in all 4 specimens; the parasite loads ranged from 18.6 to 47.9 larvae/g (Figure; Table 3). Two specimens contained a few larvae with very slight motility. Molecular testing using real-time PCR targeting the encapsulated North American *Trichinella* spp. identified the larvae as *T. spiralis* nematodes (Table 3).

**Figure:**
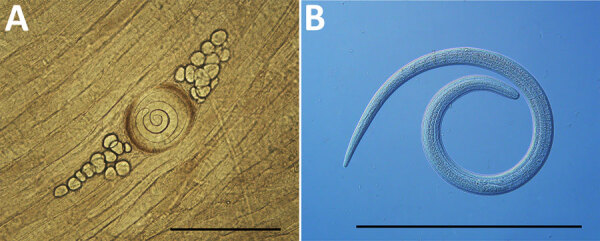
*Trichinella spiralis* larva from contaminated bear meat recovered from investigation into trichinellosis outbreak in North Carolina, USA, 2024. A) Encapsulated *T. spiralis* larva in squash prep of bear meat. B) Liberated *T. spiralis* larva after artificial pepsin-hydrochloric acid digestion shown under differential interference contrast. Scale bars equal 500 µm.

**Table 3:** Molecular and parasite load results for frozen bear meat quantified at Centers for Disease Control and Prevention from investigation into trichinellosis outbreak in North Carolina, USA, 2024

Specimen ID	Meat cut	Molecular identification*	Parasite load, larvae/g	Motile larvae
A	Tenderloin	*Trichinella spiralis*	32.7	No
B	Ham	*T. spiralis*	47.9	No
C	Ham	*T. spiralis*	46.7	Yes
D	Ham	*T. spiralis*	18.6	Yes

*Tested by using a previously published real-time PCR designed to target encapsulated North American *Trichinella* species (*5*).

## Discussion

We describe a trichinellosis outbreak in western North Carolina, providing additional evidence of changing trichinellosis patterns. Trichinellosis was rarely reported in North Carolina until 2023, when a probable outbreak of trichinellosis resulted in 10 symptomatic cases (*3*). However, the causative *Trichinella* species could not be determined. In the outbreak described in this article, *T. spiralis* nematodes were identified from leftover meat. This finding was somewhat unexpected because *T. spiralis* infection is rarely reported from bear meat, compared with rates for other *Trichinella* spp.(*6*). Wildlife disease surveillance is needed to update our knowledge of *Trichinella* prevalence, host affinities, and associated public health risks. 

The outbreak we report also underscores the need for prevention to avoid painful, debilitating infections. Despite the risk for severe disease from trichinellosis, including persistent myalgia or even death, persons preparing wild game and other potentially affected meats for consumption still neglect to follow the recommended cooking measures. Furthermore, provider awareness of trichinellosis can help enable appropriate treatment and guide public health action. Treatment of trichinellosis is typically with albendazole or mebendazole and can be supplemented with steroid drugs for severe cases (*7*).

Trichinellosis is rare in the United States. The disease was once mostly associated with pork, but changes in husbandry practices coincided with a decrease in trichinellosis cases domestically (*8*). More recently, the few cases reported each year are mostly associated with consumption of wild game meat (*8*), with bear meat being the suspected or confirmed food source for recently reported outbreaks in the United States (*9*). The trichinellosis outbreak reported in this article and the 2023 probable outbreak in North Carolina (*3*) were both linked to undercooked bear meat harvested in-state. An increasing number of bears are harvested each year in North Carolina; ≈1,500 were harvested in 2023 (*10*). In the United States and Canada, human trichinellosis outbreaks associated with bear meat are more commonly attributed to infection with *T. nativa* nematodes (*9*,*11*), whereas contemporary outbreaks of *T. spiralis* infection are mostly associated with consumption of wild boar (*6*). A report from 2003 suggested that 2 *T. nativa* trichinellosis cases in Tennessee occurred because of a bear hunted in Canada (*11*), rather than in the southeastern United States. In Canada, polar bears and grizzly bears have been confirmed to carry *Trichinella* spp. nematodes (*6*).

Trichinellosis outbreaks associated with jerky prepared from bear (*12*) and other wild game (*13*) have been reported previously. In the outbreak described in this article, jerky preparation consisted of marinade and drying steps that likely did not reach sufficient temperatures for safe consumption. Freezing wild game meat before jerky preparation is sometimes recommended as a treatment step (https://nchfp.uga.edu) but might not be sufficient for killing freeze-resistant *Trichinella* spp. nematodes, such as *T. nativa* (*7*,*9*). Although *T. spiralis* nematodes are usually considered freeze-susceptible, experimental infection trials have demonstrated persistent larval motility after several weeks of freezing, albeit with declining infectivity over time (*14*). The weak motility noted in larvae from this report is not evidence of persistent infectivity after freezing, but because of the uncertainties and potential diversity of *Trichinella* in bear meat, freezing alone cannot be assumed to reliably eliminate infection risk. Cooking meat to an internal temperature >165°F (>74°C) verified by a meat thermometer remains the best method for killing all *Trichinella* species across different types of meat. Safe food handling practices, such as processing raw or undercooked meat separately from other foods, can prevent trichinellosis spread by cross-contamination (*9*,*15*).

Trichinellosis symptoms can be severe, and diagnosis can be challenging. Serologic testing for *Trichinella* IgG is often used to diagnose trichinellosis; however, IgG seroconversion might not be detectable until weeks after infection, potentially causing false negatives if serum is collected during acute infection. Convalescent serum samples are crucial for diagnostic and public health purposes, but their acquisition remains challenging. In addition, healthcare visits, diagnostic tests, and treatment are more difficult for uninsured patients, as demonstrated by the 2 symptomatic patients who did not receive testing during this outbreak. 

In conclusion, we report an outbreak of trichinellosis in North Carolina that resulted from undercooked, locally harvested bear prepared as jerky, providing evidence of changing trichinellosis patterns. Low-cost safety measures can help prevent illness, and communication of disease prevention through safe wild game preparation is critical to avoid future outbreaks.
